# SUPPORT Tools for evidence-informed health Policymaking (STP) 8: Deciding how much confidence to place in a systematic review

**DOI:** 10.1186/1478-4505-7-S1-S8

**Published:** 2009-12-16

**Authors:** Simon Lewin, Andrew D Oxman, John N Lavis, Atle Fretheim

**Affiliations:** 1 Norwegian Knowledge Centre for the Health Services, P.O. Box 7004, St. Olavs plass, N-0130 Oslo, Norway; Health Systems Research Unit, Medical Research Council of South Africa; 2 Norwegian Knowledge Centre for the Health Services, P.O. Box 7004, St. Olavs plass, N-0130 Oslo, Norway; 3 Centre for Health Economics and Policy Analysis, Department of Clinical Epidemiology and Biostatistics, and Department of Political Science, McMaster University, 1200 Main St. West, HSC-2D3, Hamilton, ON, Canada, L8N 3Z5; 4 Norwegian Knowledge Centre for the Health Services, P.O. Box 7004, St. Olavs plass, N-0130 Oslo, Norway; Section for International Health, Institute of General Practice and Community Medicine, Faculty of Medicine, University of Oslo, Norway

## Abstract

*This article is part of a series written for people responsible for making decisions about health policies and programmes and for those who support these decision makers*.

The reliability of systematic reviews of the effects of health interventions is variable. Consequently, policymakers and others need to assess how much confidence can be placed in such evidence. The use of systematic and transparent processes to determine such decisions can help to prevent the introduction of errors and bias in these judgements. In this article, we suggest five questions that can be considered when deciding how much confidence to place in the findings of a systematic review of the effects of an intervention. These are: 1. Did the review explicitly address an appropriate policy or management question? 2. Were appropriate criteria used when considering studies for the review? 3. Was the search for relevant studies detailed and reasonably comprehensive? 4. Were assessments of the studies' relevance to the review topic and of their risk of bias reproducible? 5. Were the results similar from study to study?

## About STP

*This article is part of a series written for people responsible for making decisions about health policies and programmes and for those who support these decision makers. The series is intended to help such people ensure that their decisions are well-informed by the best available research evidence. The SUPPORT tools and the ways in which they can be used are described in more detail in the Introduction to this series *[1]. *A glossary for the entire series is attached to each article (see Additional File *1*). Links to Spanish, Portuguese, French and Chinese translations of this series can be found on the SUPPORT website *http://www.support-collaboration.org. *Feedback about how to improve the tools in this series is welcome and should be sent to: *STP@nokc.no.

## Scenarios

*Scenario 1: You are a senior civil servant and will be submitting a proposal to the Minister regarding the evidence to support a number of policy and programme options to address a priority health issue. You are concerned about how much confidence can be placed in systematic reviews of the evidence for each option and want to ensure that these have been assessed appropriately by your staff*.

*Scenario 2: You work in the Ministry of Health and are preparing a document regarding options to address a priority health issue. A number of systematic reviews of the effects of options have been identified and you have been asked to make an assessment of how much confidence can be placed in each review*.

*Scenario 3: You work in an independent unit that supports the Ministry of Health in its use of evidence in policymaking. You are preparing a document for the Ministry on the likely impacts of options to address a priority health issue. You want guidance on assessing how much confidence can be placed in the systematic reviews of the impacts of each option*.

## Background

For decision makers (Scenario 1), this article suggests a number of questions that they might ask their staff to consider when deciding how much confidence to place in the findings of a systematic review of the effects of healthcare interventions.

For those who support policymakers (Scenarios 2 and 3), this article suggests a number of questions that can be used to guide a critical appraisal of systematic reviews of effects.

Systematic reviews of randomised controlled trials (RCTs) are widely accepted as providing the most reliable evidence about the effects of healthcare interventions [2,3]. Systematic reviews are characterised by their systematic and explicit approach to identifying, selecting and appraising relevant research, and to collecting and analysing data from included studies [3]. Increasingly, systematic reviews are also being used to identify, appraise and combine evidence on the economic consequences of interventions [4], such as the cost-effectiveness of breastfeeding promotion for infants in neonatal units [5] or the costs of different guideline dissemination and implementation strategies [6]. They are also used to summarise evidence from qualitative studies, such as consumer or provider views of health interventions [7-10]. In this article, we focus on systematic reviews of the effects of healthcare policies or programmes. These include reviews of delivery arrangements, such as the effects of substituting doctors with nurses in primary care [11], and of strategies to bring about change, such as the effects of continuing education meetings for health professionals [12].

The systematic and explicit approach used in a systematic review is intended to reduce the risk of bias and errors that occur by chance, and to help facilitate critical appraisal of these syntheses [13,14]. However, the rigour with which systematic reviews are conducted varies. Reviews are therefore not all equally reliable - that is, reviews may differ in the level of confidence that we can place in their findings. Simply relying on the fact that an assessment is called a 'systematic review' (or a meta-analysis) is therefore not sufficient when using findings to inform policy decisions.

**Figure 1:**
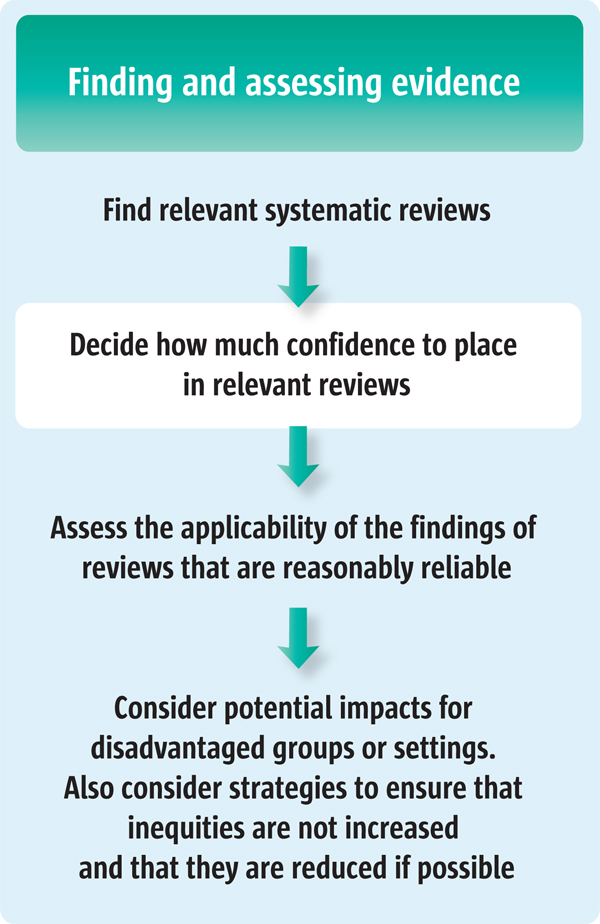
**Finding and assessing systematic reviews to inform decisions about policy and programme options**.

When using systematic reviews of effects to inform policy decisions, policymakers and others therefore need to judge how much confidence they can place in this evidence. Using a systematic and transparent process can help to prevent the introduction of errors and bias in their judgements. A systematic and transparent process also allows other stakeholders, including the public, to understand and appraise these judgements. This is particularly important where such assessments influence recommendations or decisions regarding clinical interventions or services [15], or decisions to implement or stop programmes or policies. Figure 1 outlines the steps involved in finding and assessing systematic reviews to inform policymaking.

Confidence in the findings of a systematic review may be limited for a number of reasons, including a failure to:

• Specify the question and methods of the review before undertaking the review, for example in a published review protocol

• Specify clear criteria for study inclusion and exclusion

• Adequately describe the studies included in the review

• Assess the risk of bias for studies included in the review

• Assess the risk of publication bias, i.e. the possibility that some studies, typically those with positive ('statistically significant') results, are more likely than others to be published and therefore included in a review

• Use appropriate methods for combining the results of the included studies (in a meta-analysis) where relevant

• Adequately examine differences in the findings of studies included in a review (i.e. the 'heterogeneity' of the findings)

• Base the conclusions of the review on the included data

Other potential limitations of systematic reviews include conflicts of interest (which can affect the reliability of a review in any of the ways listed above), and reviews being out-of-date.

Variations in reliability, for example, were noted in a study comparing the methodology and reporting components of Cochrane reviews with reviews published in paper-based journals. This study found that Cochrane reviews included components that made them less prone to bias. This overall reduction in the risk of bias in Cochrane reviews was found to be due specifically to both their clear descriptions of the criteria for inclusion and exclusion, and the formal assessment of the risk of bias of the studies included in each review [16]. Similarly, another study compared the methodological quality and conclusions in Cochrane reviews of drug trials with those in industry-supported reviews of the same drugs. This study found that Cochrane reviews scored higher on quality assessment. This was because Cochrane reviews considered potential for bias more frequently when compared to reviews that were industry-supported. Industry-supported reviews were also found to be significantly more likely to recommend the drugs in question without reservations [17]. A number of other studies of reviews have also reported differences in their quality and conclusions [18-21].

A number of tools have been designed to assess the quality of systematic reviews including AMSTAR (A MeaSurement Tool to Assess Reviews) [22], CASP (Critical Appraisal Skills Programme) [23], and one developed by Oxman and Guyatt [24] (also see [25,26]), but all contain similar criteria. (The AMSTAR tool is described in Table 1.) Several tools also include rating scales to score the level of confidence that can be placed in a review. Increasing numbers of reviews now include such assessments. In general, high ratings suggest that greater confidence can be placed in the findings of reviews. In contrast, low ratings indicate that less confidence can be placed in review findings and that reviews should be examined closely to identify their key limitations. Three points, though, should be noted: firstly, an overall score or rating does not necessarily indicate which particular aspects of a review were conducted reliably - some may have been conducted more reliably than others. Secondly, the scoring process itself also involves assigning weightings to different items in the assessment tool. It may be difficult to justify which items should be weighted more heavily [27]. Finally, rating tools can only assess the reliability of what is reported. When key information about the methods used in a review is not reported, it may be unclear what was done, or the extent to which what *was *done constitutes an important limitation.

**Table 1:** AMSTAR - A MeaSurement Tool to Assess Reviews (from [22])

1. Was an 'a priori' design provided? The research question and inclusion criteria should be established before the conduct of the review	□ Yes □ No □ Can't answer □ Not applicable
**2. Was there duplicate study selection and data extraction?** There should be at least two independent data extractors, and a consensus procedure for disagreements should be in place	□ Yes □ No □ Can't answer □ Not applicable

**3. Was a comprehensive literature search performed?** At least two electronic sources should be searched. The report must include the years and databases used (e.g. Central, EMBASE, and MEDLINE). Key words and/or MESH terms must be stated and, where feasible, the search strategy should be provided. All searches should be supplemented by consulting current contents, reviews, textbooks, specialised registers, or experts in the particular field of study, and by reviewing the references in the studies found	□ Yes □ No □ Can't answer □ Not applicable

**4. Was the status of publication (i.e. grey literature) used as an inclusion criterion?** The authors should state that they searched for reports regardless of their publication type. The authors should state whether or not they excluded any reports (from the systematic review), based on their publication status, language, etc.	□ Yes □ No □ Can't answer □ Not applicable

**5. Was a list of studies (included and excluded) provided?** A list of included and excluded studies should be provided	□ Yes □ No □ Can't answer □ Not applicable

**6. Were the characteristics of the included studies provided?** In an aggregated form such as a table, data from the original studies should be provided about the participants, interventions and outcomes. The ranges of characteristics in all the studies analysed e.g. age, race, sex, relevant socioeconomic data, disease status, duration, severity, or other diseases should be reported	□ Yes □ No □ Can't answer □ Not applicable

**7. Was the scientific quality of the included studies assessed and documented?** 'A priori' methods of assessment should be provided (e.g. for effectiveness studies if the author(s) chose to include only randomised, double-blind, placebo controlled studies, or allocation concealment as inclusion criteria). For other types of studies, alternative items will be relevant	□ Yes □ No □ Can't answer □ Not applicable

**8. Was the scientific quality of the included studies used appropriately in formulating conclusions?** The methodological rigour and scientific quality of the studies should be considered in the analysis and the conclusions of the review, and explicitly stated when formulating recommendations	□ Yes □ No □ Can't answer □ Not applicable

**9. Were the methods used to combine the findings of studies appropriate?** For the pooled results, a test should be done to ensure the studies were combinable and to assess their homogeneity (i.e. Chi-squared test for homogeneity, I^2^). If heterogeneity exists a random effects model should be used and/or the clinical appropriateness of combining should also be taken into consideration (i.e. was it appropriate to combine the results?)	□ Yes □ No □ Can't answer □ Not applicable

**10. Was the likelihood of publication bias assessed?** An assessment of publication bias should include a combination of graphical aids (e.g. a funnel plot, other available tests) and/or statistical tests (e.g. Egger regression test)	□ Yes □ No □ Can't answer □ Not applicable

**11. Was the conflict of interest stated?** Potential sources of support should be clearly acknowledged in both the systematic review and the included studies	□ Yes □ No □ Can't answer □ Not applicable

An *assessment *of how much confidence can be placed in the findings of a review needs to be differentiated from an *understanding *of the results of the review itself. Table 2 provides guidance on what to look for in the results of a review of effects. Guidance for assessing how much confidence can be placed in the findings of reviews of qualitative studies and of reviews of economic studies is shown in Table 3.

**Table 2:** Interpreting the results of systematic reviews of effects

The following questions can help to guide policymakers in interpreting the findings of systematic reviews of effects (adapted from [33,47,48])*:
• *What estimate of effect is presented? *Many reviews present an average estimate of effect across the included studies. This is often in the form of a risk ratio, odds ratio, or standardised mean difference
• *Is an average estimate of effect across studies appropriate? *Reviews use statistical methods to summarise and combine outcome data from the studies included in the review. To ensure that the combining of outcome data is appropriate, it is useful to consider whether the included studies were sufficiently similar in terms of population, intervention, comparison, and the outcomes measured. Where an average estimate of effect is not possible, reviews usually present a narrative overview of the available data
• *Are confidence limits for the estimate of effect presented? *The review should present confidence intervals around the average estimate of effect. The wider the confidence interval the less certain we can be about the true magnitude of the effect
• *If the results of subgroup analyses are reported, are these appropriate? *A review may present findings for a particular subgroup of participants across all trials or for a subgroup of studies [49]. For example, a review of interventions to reduce diarrhoeal diseases in children less than 5 years of age might also consider the effects of the interventions on children less than 1 year of age. Similarly, a review may include a subgroup analysis of studies judged as having a low risk of bias. A subgroup analysis should make sense in relation to both the overall review question and prior knowledge of factors that may have influenced or moderated the effects of the intervention. For example, it might be anticipated that a higher intensity intervention may produce larger effects. Subgroup analyses should be planned before a review is undertaken and less confidence should be placed in these particular results. This is because they are less reliable than analyses based on all of the included trials and because multiple statistical analyses may produce positive findings by chance alone
• *If there is 'no evidence of effect' is caution taken not to interpret this as 'evidence of no effect'? *'No evidence of effect' is not the same as 'evidence of no effect'. The former suggests that insufficient evidence is available to draw conclusions regarding the effects of the intervention in question. The latter suggests that there is clear evidence from the included studies that the intervention does not have the anticipated effects [50]
• *Do the conclusions and recommendations (if any) flow from both the original review question and the evidence that is presented in the review? *It is important to consider whether the conclusions presented by the review authors emerge directly from the data gathered from the review and do not go beyond this evidence
• *Is the evidence applicable to the policy question under consideration? *Differences in health systems can mean that a programme or intervention that works in one setting may not work the same way in another. Policymakers need to assess whether the research evidence from a review applies in their setting. Guidance on this is presented in Article 9 in this series [28]
* There is some overlap between the questions listed here and those intended to guide assessment of the reliability of systematic reviews. This is because reliability is an important element in assessing and understanding the results of a systematic review

**Table 3:** Assessing how much confidence can be placed in the findings of systematic reviews of qualitative studies and systematic reviews of economic studies

An increasing number of systematic reviews of qualitative studies are being undertaken. These use a wide range of approaches, including narrative synthesis, meta-ethnography and realist review. As well as providing important information in their own right, reviews of qualitative studies can also inform and supplement systematic reviews of effects [51,52]. However, it is important for the reader to assess the reliability of these reviews. To date, few tools have been designed for this specific purpose. Many of the questions used to guide policy makers when assessing the reliability of systematic reviews of effects, however, are also useful for reviews of qualitative studies. These include:
*1. Did the review address an appropriate policy or management question? *The review question should be amenable to being addressed using qualitative data and should be relevant to policymaking. Reviews of qualitative studies can provide insights about stakeholders' views and experiences regarding health and healthcare and thus help to clarify a problem [39]. Reviews of qualitative studies can also provide information on how or why options work (for example, through examining process evaluations conducted alongside the implementation of a policy or programme) and about stakeholders' views about the options and their relevant experiences [40,53]
*2. Were the criteria used to select studies appropriate? *The description of how studies were selected should be appropriate in relation to the research question
*3. Was a clear and appropriate explanation provided for the search approach used? *Some reviews of qualitative studies undertake comprehensive literature searches while others may use sampling approaches. The chosen approach should be clearly described and justified
*4. Was the approach used to assess the reliability of the included studies appropriate? *The review should describe how the reliability of the included studies was taken into account
*5. Was an appropriate approach used to analyse the findings of the included studies? *The review should use an accepted approach to synthesis and should describe the rationale for the approach chosen

**Questions to consider when assessing the reliability of reviews of economic studies include (from **[54]**):**
*1. Is it unlikely that important relevant studies were missed?*
*2. Were the inclusion criteria used to select articles appropriate?*
*3. Was the assessment of studies reproducible?*
*4. Were the design and/or methods and/or topic of included studies broadly comparable?*
*5. How reproducible are the overall results?*
*6. Will the results help resource allocation in healthcare?*

An assessment of the degree of confidence that can be placed in review findings also needs to be differentiated from any assessment that might be done of the *relevance *of reviews to particular policy questions. Considerations of relevance include, for example, questions related to whether a review provides evidence of the effects of the different policy or programme options under consideration, and whether the findings of a review are applicable to the setting in which the policy will be implemented. The process of assessing the applicability of the findings from systematic reviews is discussed further in Article 9 in this series [28].

In this article, we suggest five questions that can be considered when deciding how much confidence to place in the findings of systematic reviews of the effects of options.

## Questions to consider

The following questions can guide policymakers when deciding how much confidence to place in the findings of a systematic review of the effects of an option:

1. Did the review explicitly address an appropriate policy or management question?

2. Were appropriate criteria used when considering studies for the review?

3. Was the search for relevant studies detailed and reasonably comprehensive?

4. Were assessments of the studies' relevance to the review topic and of their risk of bias reproducible?

5. Were the results similar from study to study?

### 1. Did the review explicitly address an appropriate policy or management question?

A key first step in assessing the confidence that can be placed in the findings of a systematic review is to examine the *question *that is being addressed. The technical design and conduct of a review may well be excellent, but the findings of a review are unlikely to be useful in decision making if they have not explicitly addressed a policy or management question that is sensible, appropriate and relevant to the issue that a policymaker is considering.

An appropriate policy or management question will:

• *Be explicit*: in other words, it will be stated in detail rather than implied in the material presented. If the review question was not expressed explicitly or formulated clearly, it is difficult to assess the conduct of the review adequately. This is because the conduct of the review will need to be considered, at least in part, in relation to the question itself [29]. For example, an appraisal of whether the criteria used to select studies for a review were appropriate, needs to be done in relation to the review question that the studies were intended to answer. A clear question also helps readers to assess whether a review is relevant to their work [29]

• *Be established a priori*: in other words, *before *the review was conducted. It is important that the review question be specified before a review is conducted, preferably in a review protocol or plan. All Cochrane reviews, for example, are preceded by a published review protocol and examples of these can be found in the Cochrane Library http://www3.interscience.wiley.com/cgi-bin/mrwhome/106568753/HOME. If the review question is not specified before the review is conducted, there is a risk that the question may have been altered to suit the evidence found, thus undermining confidence in the findings

• *Address a question of relevance to policymaking or management*. This will need to be assessed in a specific context, based on the range of issues that are important in a particular jurisdiction at a particular time. A review question may not be relevant if:

◦ *It is too narrow*: for example, a review may consider the effects of a programme on a specific age group of participants only, located in a particular setting, or for a restricted range of outcomes. It would not be possible, in this instance, to generalise the results to other populations, settings or outcomes

◦ *It is too broad*: a review, for example, may define a programme as including a very broad range of practices and not all of these may be relevant to a particular jurisdiction. Or a review may pose a very broad question that is not useful from a decision-making perspective. A question such as whether nurses can effectively deliver health promotion programmes, for instance, will not be useful in deciding whether a particular cadre of nurses, such as enrolled nurses, can effectively deliver a health promotion programme for a specific health issue, such as HIV/AIDS prevention

◦ *It does not specify an appropriate comparison group*: if, for example, a programme is compared to a 'no programme' scenario rather than to current best treatment for a condition

A well-formulated review question should specify *all *of the following: the types of population and settings that the review will cover (e.g. children aged between one month and six years of age living in a malaria-endemic area); the types of programmes and comparisons considered (e.g. anti-malarial drugs given at regular intervals (the intervention) compared to placebo or no drug (the comparison)); and the types of outcomes that are of interest (e.g. clinical malaria and severe anaemia) [30,31]. The acronym PICO (Population, Intervention, Comparison, Outcomes) is sometimes used to summarise these four key components of a review question.

While the need for a well-formulated review question may seem obvious, many narrative reviews fail to provide this. A review of a sample of such reviews published in major medical journals showed that 20% failed to state their purpose clearly [32].

### 2. Were appropriate criteria used when considering studies for the review?

Inclusion and exclusion criteria for a review are the detailed listings of the types of population, interventions, comparisons and outcomes that a review will consider. These criteria, specified in a review protocol, will determine which studies are included in a review. They will therefore influence strongly the findings of a review. It is important that these criteria are appropriate in relation to the review question.

The following questions should be examined when considering whether the criteria used to consider studies for a review are appropriate:

• *Does the review specify clear inclusion and exclusion criteria*? These criteria are important as a way of protecting against bias related to the inclusion of studies in the review. A recent assessment of the methodological quality of systematic reviews in general surgery, for example, found that only 70% of these reported the criteria used for deciding which studies to include in a review [18]

• *Are the inclusion and exclusion criteria explicit* in relation to the following: the types of population considered, the types of interventions and comparisons considered, and the types of outcomes considered?

• *Are the inclusion and exclusion criteria congruent with the review question*? [33] For example, if a review aims to evaluate prophylaxis and intermittent treatment with anti-malarial drugs to prevent malaria in young children living in malaria-endemic areas, do the criteria indicate the inclusion of studies of children from the appropriate settings, and do they specify the forms of prophylaxis and treatment that will be considered? [31] Similarly, if a review aims to examine the effects of interventions to increase the proportion of health professionals working in rural and other underserved areas, do the criteria indicate the range of healthcare professionals that will be included and the types of educational or financial interventions that will be considered? [34]

### 3. Was the search for relevant studies detailed and reasonably comprehensive?

A key aspect of a systematic review is a thorough and reproducible search of the literature for studies that meet the eligibility criteria of a review. This approach is one of the elements that differentiates *systematic *reviews from *narrative *reviews. Systematic searching contributes to minimising bias in a review by ensuring that all relevant evidence is considered. It therefore helps to achieve reliable estimates of the effects of the policy or programme being examined [35].

Publication bias - that is, the selective publication of studies based on the direction and strength of their results [36] - is one route by which bias may be introduced into reviews. A recent review examined the extent to which the publication of randomised trials is influenced by whether or not positive results were found and the perceived importance of trial findings. It showed that trials with positive results were significantly more likely to be published than trials that presented negative findings [37]. This review and other research also showed that trials reporting positive findings are published sooner than others [38]. As a result, reviews may overestimate the positive effects of programmes unless attempts are made to identify both published and unpublished studies.

Systematic reviews vary in the extent to which they include comprehensive searching. A review of the reporting of published reviews on the treatment of asthma, for example, found that only 52% of the 33 examined reviews included a reasonably comprehensive search for evidence of effects [20]. It is therefore important to check how searches for relevant studies were conducted.

The following questions should be examined when considering whether the search for relevant studies was detailed and reasonably comprehensive [22]:

• *Does a review describe in detail the strategy used to search for relevant studies?* This reporting should include: 1. The list of sources searched, 2. The key words used to search these sources (where applicable), and 3. The years over which the sources were searched. Table 4 provides examples of the range of sources searched in reviews published in the Cochrane Library

**Table 4:** Examples of sources searched in systematic reviews

Review	Sources searched
*Health systems review* Example: Systematic review of lay health worker interventions in primary and community healthcare [44]	1. Electronic databases of published studies:
	• MEDLINE
	• Cochrane Central Register of Controlled Trials (CENTRAL) and specialised Cochrane Registers (EPOC and Consumers and Communication Review Groups)
	• Science Citations
	• EMBASE
	• CINAHL (Cumulative Index to Nursing and Allied Health Literature)
	• Healthstar
	• AMED (Allied and Complementary Medicine Database)
	• Leeds Health Education Effectiveness Database
	
	2. Bibliographies of studies assessed for inclusion
	
	3. All contacted authors were asked for details of additional studies

*Public health review* Example: Systematic review of male circumcision for prevention of heterosexual acquisition of HIV in men [55]	1. Electronic databases of published studies:
	• MEDLINE
	• EMBASE
	• Cochrane Central Register of Controlled Trials (CENTRAL)
	
	2. Electronic databases of conference abstracts:
	• AIDSearch Conference databases
	
	3. Electronic databases of ongoing trials:
	• ClinicalTrials.gov
	• Current Controlled Trials
	
	4. Contacted researchers and relevant organisations in the field
	
	5. Checked the reference lists of all studies identified by the above methods and examined any systematic reviews, meta-analyses, or prevention guidelines identified during the search process

*Clinical review* Example: Systematic review of statins for the prevention of dementia [56]	1. Electronic databases:
	• The Specialized Register of the Cochrane Dementia and Cognitive
	• Improvement Group
	• Cochrane Central Register of Controlled Trials (CENTRAL)
	• MEDLINE
	• EMBASE
	• PsycINFO (a database of psychological literature)
	• CINAHL
	• SIGLE (Grey Literature in Europe)
	• LILACS (Latin American and Caribbean Health Science Literature)
	
	2. Electronic databases of conference abstracts:
	• ISTP (Index to Scientific and Technical Proceedings)
	• INSIDE (British Library Database of Conference Proceedings and Journals)
	
	3. Electronic databases of theses:
	• Index to Theses (formerly ASLIB) (United Kingdom and Ireland theses)
	• Australian Digital Theses Program
	• Canadian Theses and Dissertations
	• DATAD - Database of African Theses and Dissertations
	• Dissertation Abstract Online (USA)
	
	4. Electronic databases of ongoing trials: searched a large range of such databases

• *Did the search strategy include electronic databases of published studies*? A wide range of electronic databases of published studies is available and several can be searched at no or very low cost. Key databases include PubMed/MEDLINE (compiled by the National Library of Medicine, USA), the Cochrane Central Register of Controlled Trials (CENTRAL - compiled by the Cochrane Collaboration), and regional databases such as LILACS (Latin American and Caribbean Health Sciences). Articles 4 [39] and 5 [40] in this series provide further information on finding relevant research literature

• *Were the searches of electronic databases supplemented by additional searching?* This might have included an examination of the reference lists of relevant studies, making contact with authors and experts in the field, and the consultation of specialised registers of studies related to the topic area of the review. This additional searching is useful as a way of helping to identify both further published studies and unpublished studies (which may include studies available in the 'grey' literature, i.e. in sources of literature other than indexed, peer-reviewed journals)

• *Are the searches up-to-date?* Does the review specify the period covered by the searches and are the searches current? A published review, while relevant to a policy question, may have used searches that are now several years old. It is therefore possible that the review does not include all the latest relevant evidence and may therefore give an unreliable estimate of the effects of the policy or programme option

### 4. Were assessments of the studies' relevance to the review topic and of their risk of bias reproducible?

Authors of systematic reviews need to make two important judgements regarding each primary study that might be included in a review. Firstly, does the study meet the criteria for inclusion in their review - in other words, is it relevant to the review topic? Secondly, what is the risk of bias in the results of the study? Risk of bias refers to the risk of "a systematic error, or deviation from the truth, in results or inferences" [27]. It also relates to the question of whether the results of a study can be assumed to be accurate [27]. Because these judgements will affect the findings of a review, it is important that they are presented in a way that is transparent and reproducible. Others need to be able to understand how these judgements were made and to be able to repeat these assessments.

As discussed above, reviews need to specify clear inclusion and exclusion criteria in order to protect against bias in the process of selecting studies for inclusion. These criteria and judgements will necessarily affect the findings of the review by influencing the studies selected for inclusion. Bias or errors in these judgements can be minimised in the following ways: firstly, two reviewers should decide independently on which studies to include in a review. Additional discussions with other reviewers can also be used to resolve disagreements related to the inclusion of a particular study. Secondly, reasons for the inclusion of a study (and for excluding a study that appears relevant) should be recorded in the published review. This will allow readers to make their own judgements regarding eligibility decisions. It also provides a transparent 'audit trail' for the review, ensuring that the process is reproducible.

The ability of a systematic review to reach conclusions regarding the effects of a policy or programme also depends on the validity of the data obtained from each included study. Pooling the results of the studies, or creating a summary of them in a review, may give a misleading result if the validity of the individual studies included in the review is low. Evaluating the risk of bias in the results of the included studies is therefore an important element of a systematic review. Such assessments should feed into the interpretation and conclusions of a review [27].

A number of different approaches for assessing quality or risk of bias have been developed for randomised trials [27,41,42]. While we do not discuss these different approaches here, it is important to note that reviews should be explicit regarding the approaches used and should apply these consistently.

When assessing the relevance of the included studies to the review topic and the potential risk of bias, the following questions should be considered:

• *Was an explicit and transparent approach used to assess the relevance of studies to the review topic?* A review should state how relevance was assessed and provide a list of both included and excluded studies

• *Was an explicit and transparent approach used to assess the risk of bias in the included studies?* A review should report the tool used to assess the risk of bias, how the assessment was conducted, and the results of the assessment

•* Were the results of the risk of bias assessment taken into account in interpreting the results of a review? *When the risk of bias in the included studies is high, for example, we might have less confidence in the findings of a review

### 5. Were the results similar from study to study?

The findings of the studies included in a review may be very similar - or they may vary - in terms of the effects of the programme on a particular outcome. This variability among the studies included in a review is usually referred to as 'heterogeneity' [27]. The variability among studies included in a review depends in part on the scope of the review. Where the scope is wide, the range and therefore the variability of the included studies might also be expected to be wide. In contrast, where the scope of a review is narrow, the included studies are likely to be more similar to each another.

If the participants, interventions or outcomes of the studies included in a review are very different, this may lead to variation or heterogeneity if the intervention effect is affected by these factors. Because the true intervention effect will be different across these studies, in these instances the average effect across the studies will not be helpful.

Depending on the level of variability, reviews may use different approaches to summarising information from the studies included, for example:

• *Calculating the average (or pooled) effect across studies*: this approach is useful when the variability across studies is low. For example, a systematic review of 'early hospital discharge combined with hospital at home' programmes (i.e. programmes in which active treatment is given by health providers in a patient's home for a health issue that would otherwise require acute hospital inpatient care) found that the studies included were sufficiently similar to be able to estimate the average effect of the programme. The review found insufficient evidence of economic or health benefits from 'early discharge hospital at home' programmes [43]

• *Calculating the average effect for subgroups of studies included in a review*: this may be useful when the overall variability of studies included in a review is high (and it is therefore unhelpful to calculate an average affect), but where variability is low among subgroups of studies. For example, a review of lay health worker interventions in primary and community healthcare grouped studies according to the health issues addressed by the lay health workers. For some of the groups, such as lay health workers to promote immunisation and breastfeeding, it was possible to calculate an average effect across the relevant studies. The review found evidence that lay health workers can improve immunisation and breastfeeding uptake [44]

• *Describing the range of effects sizes*: where studies are not sufficiently similar to make calculating an average effect useful, it may still be possible to describe the range of effects found in the studies. For example, a review of the effects of audit and feedback on the practice of healthcare providers showed that compliance with desired practice ranged from a decrease of 16% to an increase of 70%, with a median of 5%. The review indicated that audit and feedback can make practice more effective but that the effects are generally small to moderate [45]

• *Cataloguing the types of interventions to address a particular issue*: the wide scope of some reviews, and therefore the variability of the studies within them, means that it is not sensible to attempt to quantitatively combine the findings of the included studies - or even to describe the range of effect sizes. In these cases, a narrative review can be undertaken. For example, a systematic review of the effectiveness of health service interventions aimed at reducing inequalities in health included studies that assessed programmes designed to reduce inequalities in health and that could be implemented within the health system alone, or in collaboration with other agencies. The range of included studies was large, extending from programmes to improve control of blood pressure, through to health promotion interventions. No statistical pooling was therefore attempted [46]

Where results differ from study to study, the following questions should be considered:

• *Is there a compelling explanation for the differences that were found? *This might include differences in the participants, interventions, comparison groups, outcomes, settings or time periods across the included studies. For example, some studies may have included participants who had a wider age range or different pre-existing health conditions

• *If a pooled estimate was made, is this likely to be meaningful? *If the studies included in a review are varied, a pooled estimate may not be meaningful. Further exploration of the data, through subgroup analysis, may be conducted but the results of such exploratory analyses may not be reliable

As the number of available systematic reviews increases, it is becoming more common to find more than one systematic review for a particular policy question. Sometimes the results or conclusions of these reviews may be different. Table 5 provides guidance on how policy makers might approach such situations.

**Table 5:** What should policymakers do when different systematic reviews that address the same question have different results?

When looking for evidence to inform a particular policy decision, it is not uncommon to identify more than one relevant systematic review. Sometimes the results of these reviews may be different, and this may result in review authors drawing different conclusions about the effects of an intervention. This scenario differs from one in which the findings of two or more reviews agree but in which researchers or others disagree on the interpretation of these findings [19]. There are many reasons why the results of different systematic reviews may differ. These include differences in: the questions addressed by the reviews, the inclusion and exclusion criteria used, which data were extracted from the studies, how the quality of the studies was assessed, and decisions regarding (and methods for) statistical analysis of the data [19].
The following series of questions designed by Jadad and colleagues can be used to assist with identifying and addressing the causes of discordance [19]:
• Do the reviews address the same question? If not, the review that is chosen should be the one which addresses a question closest to that of the policy question for which evidence is needed. Alternatively, it should assess outcomes most relevant to the policy question
• If the reviews address the same question, do they include the same trials or primary studies? If they do not include the same trials, the review that includes studies most relevant to the policy question being considered should be selected
• If the reviews include the same studies, are the reviews of the same quality? If not, the higher quality review should be used
Where both reviews are relevant, for example where they address different aspects of the same question, it may be useful to draw evidence from both.

## Conclusion

Variations are evident in the rigour with which systematic reviews of effects are conducted. It is therefore important to assess the reliability of reviews used to inform policy decisions, in order to be able to judge how much confidence can be placed in this evidence. A systematic and transparent approach to such assessments should be used and a number of tools have been developed for this purpose. However, these tools can only be used to assess what is reported. This is why any assessments that are made using these tools need to be undertaken carefully and thoughtfully.

**Figure 2:**
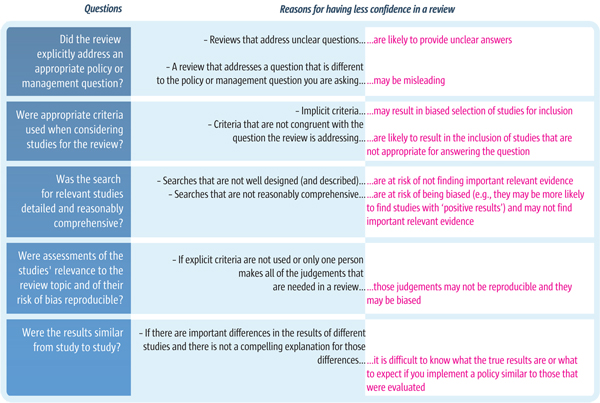
**Ways in which reviews may be unreliable and misleading**.

Where the reliability of a systematic review is poor, policymakers should have less confidence in the findings and should be cautious if using them to inform policy decisions (as summarised in Figure 2). When making decisions informed by the evidence presented in a review, policymakers need to consider assessments of the reliability of a review alongside other information, such as the usefulness of the review in relation to the policy question and evidence on the local context.

## Resources

### Useful documents and further reading

- Higgins JPT, Altman DF: **Chapter 8: Assessing risk of bias in included studies**. In *Cochrane Handbook for Systematic Reviews of Interventions Version 5.0.1 (updated September 2008)*. Edited by Higgins JPT, Green S. The Cochrane Collaboration; 2008. Available at:http://www.cochrane-handbook.org

- Counsell C: **Formulating Questions and Locating Primary Studies for Inclusion in Systematic Reviews**. *Ann Intern Med *1997, **127: **380-387

- Shea BJ, Grimshaw JM, Wells GA, Boers M, Andersson N, Hamel C *et al*.: **Development of AMSTAR: a measurement tool to assess the methodological quality of systematic reviews**. *BMC Med Res Methodol *2007, **7: **10. Available at: http://www.biomedcentral.com/1471-2288/7/10

### Links to websites

- The Rx for Change database:http://www.cadth.ca/index.php/en/compus/optimal-ther-resources/interventions - This summarises current research evidence about the effects of strategies to improve drug prescribing practice and drug use. This database includes summaries, including reliability assessments, of systematic reviews that evaluate the effects of strategies targeting professionals, the organisation of healthcare, and consumers.

- Cochrane Effective Practice and Organisation of Care (EPOC) Review Group:http://www.epoc.cochrane.org/en/index.html - The Review Group provides guidance on assessing the reliability of different types of studies of effectiveness.

- The SUPPORT (SUPporting POlicy relevant Reviews and Trials) Collaboration:http://www.support-collaboration.org/index.htm - This project produces summaries of high priority reviews for low- and middle-income countries. These include assessments of reliability.

## Competing interests

The authors declare that they have no competing interests.

## Authors' contributions

SL prepared the first draft of this article. ADO, JNL and AF contributed to drafting and revising it.

## Supplementary Material

Additional file 1 Glossary Click here for file
